# Making the Most of Complexity to Create Opportunities: Comprehensive Genomic Profiling and Molecular Tumor Board for Patients with Non-Small Cell Lung Cancer (NSCLC)

**DOI:** 10.3390/cancers13040609

**Published:** 2021-02-04

**Authors:** Caterina Fumagalli, Elena Guerini-Rocco, Massimo Barberis

**Affiliations:** 1 Division of Pathology, IEO, European Institute of Oncology, IRCCS, 20141 Milano, Italy; elena.guerinirocco@ieo.it (E.G.-R.); massimo.barberis@ieo.it (M.B.); 2 Department of Oncology and Hemato-Oncology, University of Milan, 20122 Milan, Italy

Personalized cancer therapy matches the plan of treatment with specific molecular alterations. This is the result of pivotal advances in our knowledge of tumor biology focused on the mechanisms underpinning uncontrolled tumor growth, evasion from host immune—surveillance, cell survival, migration, and the ever-increasing number of available targeted and immune therapies.

Nowadays, next-generation sequencing (NGS) is widely used for the molecular profiling of solid tumors. Over the past few years, this multigene approach has proved to be winning over a single gene sequential strategy. In many academic centers, NGS can offer a comprehensive genomic profile (CGP) to identify all four classes of alterations (base-pair substitutions, copy number variations, insertions/deletions, and rearrangements) in hundreds of genes. The NGS approach is particularly useful in NSCLC, where the number of actionable gene alterations is high [1,2] and the available histological or cytological samples can be scarce [3,4]. The National Comprehensive Cancer Network (NCCN) guidelines recommended broad molecular profiling in metastatic NSCLC, including *BRAF*, *ERBB2* (*HER2*), *MET*, *RET*, *NTRK* and *ROS1* in addition to *EGFR* and *ALK* analysis [5]. Moreover, predictive biomarkers of response to immunotherapy are essential, beyond testing for tumor programmed death-ligand 1 (PD-L1) expression [6]. Recently, tumor mutational burden (TMB), defined as the total number of mutations per coding area of the tumor genome, has been evaluated in different solid tumors [7]. However, TMB has technical and clinical limitations, and despite an initial success from clinical trials, its role as a biomarker for immunotherapy is still uncertain [8,9].

In this continuously evolving perspective, the implementation of large NGS panels is the most pragmatic way to investigate clinically useful molecular markers simultaneously. CGP can detect concurrent or uncommon alterations in frequently mutated genes, reflecting tumor genomic heterogeneity and complexity. Studies from different centers reported successful implementation of CGP [10,11,12]. In the Netherlands, the DRUP study (NCT02925234) and the TAPUR study in the United States (NCT 0269353) evaluated the outcome of patients treated with targeted therapies based on specific molecular alterations. The TAPUR study stemmed from the recognition that the rapid dissemination of genomic profiling offers an opportunity to learn from the application of precision cancer medicine in practice providing a framework for clinical decision support [13]. Indeed, CGP may produce a volume of molecular data that is tricky to manage and difficult to transpose into clinical indications. Clinical decision-making can be particularly complex for patients with advanced NSCLC, considering the number of actionable molecular aberrations and available targeted therapies. Different thorny questions could emerge: how to face tumor heterogeneity, which alterations might induce tumor resistance, or which mutation is the driver genomic aberration to be targeted first.

In this scenario, a multidisciplinary molecular tumor board (MTB) represents a valuable tool to support patient’s physicians to foster precision medicine strategies. MTB is a dynamic team, gathering professionals from different specializations, including oncologists, pathologists, molecular biologists, geneticists, radiotherapists, surgeons, translational scientists, pharmacologists, and bioinformaticians. In these meetings, all the information regarding the patient’s history, imaging, histopathology, laboratory results, and CGP are summarized and reviewed. CGP results can also be evaluated by experts using cancer-related databases [14,15,16]. However, the large-scale sequencing of tumors and the exponential growth of bioinformatics data make their interpretation complicated, prefiguring a gap between the knowledge and the implications of genomics in therapeutic planning. Moreover, more than three million peer-reviewed publications are related to cancer and more than 250 US FDA-approved oncology drugs make keeping up to date an insurmountable task.

To overcome these gaps, dedicated artificial intelligence (AI) tools are necessary [17]. AI offers a review of relevant data and evidence, matches patients’ clinical and molecular data to clinical trials avoiding a manual screening that can result in a low percentage of eligibility [18]. These systems allow a strict collaboration among specialists and increases workflow efficiency guaranteeing a comprehensive patient overview and tracking MTB decisions and follow-up. In addition, a cloud-based virtual molecular tumor board (VMTB) platform has been recently developed, with scoring models for ranking cancer treatments, allowing specialists to discuss selected cases in a time- and location-independent way [19]. Moreover, a molecular tumor board portal (MTBP) with automated NGS data interpretation and reporting, and virtual meeting, has been introduced for unifying the CGP result management across seven European cancer centers within the Cancer Core Europe (CCE) network [20]. With these supports, MTB can critically discuss the results exposed by a coordinator/navigator with the final aim to find the best match among clinico-pathological characteristics, genomic alterations and available targeted therapies. Other issues to be addressed could be the need for additional medical examinations or recommendations of genetic counseling (Figure 1).

The implementation of MTB has been recently reported as a valuable clinical tool in different scenarios [21,22,23,24,25,26]. In particular, according to the 4-year experience of West Cancer Center and Research Institute (Memphis, TN, USA), MTB expressed 837 recommendations, including the administration of standard therapy (37%), clinical trial participation (31%) and off-label therapy use (10%) [21]. In a timeframe of 3 years, the MTB at the Institute Curie in Paris (France) enabled the inclusion of 10% of patients into a clinical trial with matched therapy [23]. According to the Johns Hopkins-MTB recommendations (Baltimore, Maryland, United States), 43% of patients receiving a genomically matched therapy derived a clinical benefit lasting at least 6 months [25]. The University Medical Center in Groningen (the Netherlands) reported that MTB indications resulted in a positive clinical response in the majority (81%) of patients affected by metastatic NSCLC with rare or complex mutational profiles, achieving an objective response rate of 67%, with a median progression-free survival of 6.3 months and overall survival of 10.4 months [22]. However, drug accessibility may represent a crucial barrier, thus, clinical and hospital pharmacists should be involved in MTB to facilitate drug administration, even in expanded access programs or in compassionate use. Indeed, only by receiving personalized treatments can the improvement of patient survival associated with the CGP approach be achieved [27,28,29].

In conclusion, objective data are consistent with the indication that patients with advanced/metastatic disease often have distinct molecular alterations that may require matched treatments rather than standard therapies derived from non-biomarker-based populations [29,30]. In this setting, molecular tumor boards can be valuable tools to support a patient’s tailored treatment.

## Figures and Tables

**Figure 1:**
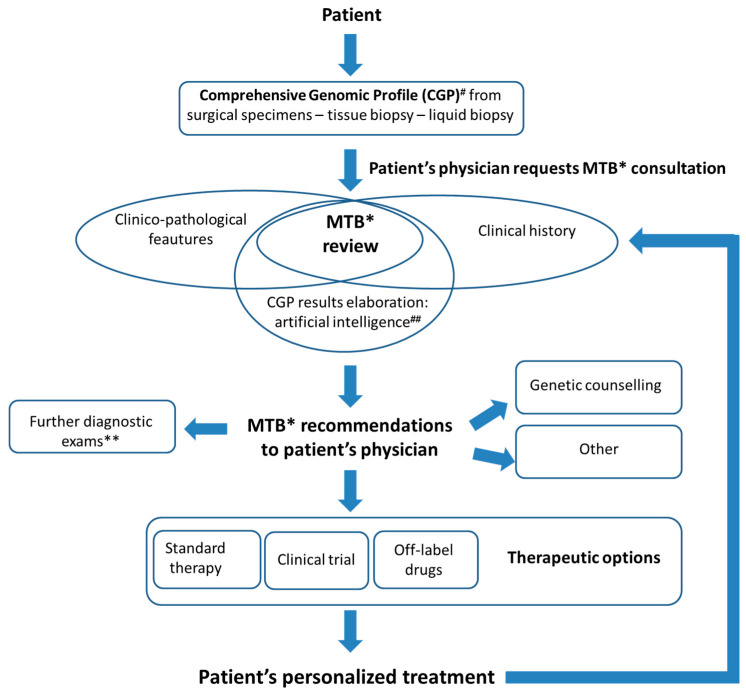
The Molecular Tumor Board (MTB) at European Institute of Oncology: the way to precision medicine. The MTB plays a pivotal role to integrate patient’s clinico-pathological features with tumor molecular data derived from comprehensive genomic profiling, taking advantage of artificial intelligence systems. The main goal is to offer to patient’s physician recommendations regarding the best—patient-tailored—therapeutic options. ^#^ Comprehensive genomic profiling performed with Oncomine Comprehensive Assay v.3 (Thermo Fisher Scientific, Waltham, MA, USA) or FoundationOne CDX (Foundation medicine—Roche) [31]. ^##^ Artificial intelligence support: Navify (Roche Diagnostics). * MTB: Molecular Tumor Board. Multidisciplinary team of specialists including oncologists, surgeons, pathologists, biologists, bioinformatics experts, geneticists, basic and translational scientists, radiologists. ** Diagnostic exams including radiologic exam, re-biopsy, histopathological revision or additional molecular tests.
